# Mapping total microbial communities and waterborne pathogens in household drinking water in China by citizen science and metabarcoding

**DOI:** 10.3389/fmicb.2025.1609070

**Published:** 2025-08-04

**Authors:** Xinyi Wen, Chutong Fang, Lihan Huang, Jiazheng Miao, Yajuan Lin

**Affiliations:** ^1^Division of Natural and Applied Science, Duke Kunshan University, Kunshan, China; ^2^Nicholas School of the Environment, Duke University, Durham, NC, United States; ^3^Department of Civil and Environmental Engineering, Pratt School of Engineering, Duke University, Durham, NC, United States; ^4^Blum Center for Developing Economies, University of California, Berkeley, CA, United States; ^5^Department of Civil and Environmental Engineering, School of Engineering, Stanford University, Stanford, CA, United States; ^6^Department of Biomedical Informatics, Harvard Medical School, Harvard University, Boston, MA, United States; ^7^Department of Life Sciences, College of Science, Texas A&M University-Corpus Christi, Corpus Christi, TX, United States

**Keywords:** citizen science, toolkit, household drinking water, microbial communities, waterborne pathogens, metabarcoding

## Abstract

**Introduction:**

Access to safe drinking water remains a critical public health priority, as waterborne diseases continue to pose global health risks. In China, microbial contamination in household water supplies is of particular concern. Traditional culture-based monitoring methods are limited in sensitivity and scope, and scaling such efforts nationwide would demand significant resources. Comprehensive, culture-independent microbiome assessments are therefore needed to better characterize microbial risks in tap water.

**Methods:**

To address this gap, we developed a cost-effective, citizen science-based approach for monitoring the tap water microbiome. Between December 2020 and August 2021, 50 household tap water samples were collected by volunteers across 19 provinces and regions in China, including several samples obtained before and/or after extreme weather events including the 2021 Henan Floods and Typhoon In-Fa. A low-biomass sampling protocol was developed and adopted, and DNA was extracted and analyzed via 16S rRNA gene metabarcoding targeting the V4 region.

**Results:**

Of the 50 samples, 22 were successfully amplified and yielded DNA with a significant number of sequencing reads. High-throughput amplicon sequencing identified 7,635 Amplicon Sequence Variants (ASVs), revealing a diverse microbiome in household tap water. Opportunistic pathogens, including *Mycobacterium*, *Acinetobacter*, and *Legionella*, were detected in all PCR-positive samples. Alarmingly, post-typhoon samples from Changzhou showed a marked increase in the relative abundance of *Escherichia coli*.

**Discussion:**

Although based on a limited number of sequenced samples, this study highlights potential microbial risks in household tap water, particularly following extreme weather events. The presence of multiple opportunistic and potentially pathogenic taxa underscores the limitations of traditional indicator-based monitoring. Our findings demonstrate the feasibility and scalability of citizen science for microbial water quality survey, offering a complementary tool for national monitoring and informing future public health strategies for water safety.

## 1 Introduction

With the growing demand for safe drinking water, microbial contamination in water resources and the related diseases it causes remain a critical concern for global water quality management. The widespread presence of human pathogens and toxin-producing bacteria in both ambient and drinking water is a well-recognized public health concern worldwide (Pandey et al., 2014). Numerous studies have shown that drinking water with microbial contamination can cause both acute and chronic health effects, contributing to a significant global burden of waterborne human diseases, including rising incidence of potentially fatal illnesses such as gastrointestinal illness (e.g., diarrhea) and liver cancers (Zhang et al., 2010; Yu et al., 2012; Wen et al., 2020; Ramirez-Castillo et al., 2015; Motlagh and Yang, 2019). Globally, more than 3.5 million deaths annually are attributed to waterborne pathogens, and one in three people still lacks access to safe, consumable water (American Society for Microbiology, 2025). Moreover, environmental changes, such as extreme weather events, rising temperatures, and aging infrastructure, are exacerbating water quality challenges. Events like floods, droughts, and typhoons increase the likelihood of pathogen intrusion into drinking water systems (American Society for Microbiology, 2025). Therefore, the global scarcity of water resources is exacerbated by contamination risks stemming from both natural and anthropogenic stressors (Yu et al., 2012; Wen et al., 2020; Wang et al., 2021).

A systematic review of China’s drinking water sanitation from 2007 to 2018 shows that microbial contamination in drinking water is a particular concern in China (Wang et al., 2021). To assess the potential health risks and support microbiological water safety management in China, water monitoring is urgently needed, especially at the point of use (Altenburger et al., 2019). China CDC (Centers for Disease Control and Prevention) at all levels sample drinking water twice a year to obtain copious water quality data (Wang et al., 2021). Due to the vastness of China, this nationwide water monitoring requires considerable investments of capital, time, personnel, and technology (Wang et al., 2021). Fortunately, previous research has demonstrated that citizen science can be an effective tool for increasing the spatial and temporal coverage of data (Pocock et al., 2017). In the context of China, citizen science could be a cost-effective approach to supplement China’s national professional water monitoring systems (Albus et al., 2019; European Commission, 2020).

Citizen science can be broadly defined as a scientific approach in which the public (i.e., people who have limited knowledge and skill in the targeted field) participate in the generation of scientific knowledge (European Commission, 2020; Brouwer et al., 2018; Roy et al., 2012; Harper, 2018), commonly in data collection (European Commission, 2020; Brouwer et al., 2018; Roy et al., 2012; Harper, 2018). Citizen science has a history spanning several centuries in Western societies, particularly in the environmental domain, which is immense (Albus et al., 2019; European Commission, 2020; Brouwer et al., 2018). Among citizen-based environmental monitoring programs, water resources monitoring is one of the major emerging fields (Albus et al., 2019). It is especially active in Western countries (Roy et al., 2012; Baalbaki et al., 2019; Buxton et al., 2018; Ho et al., 2020) since the provision of safe drinking water is a defining aspect of a developed country (Ashbolt, 2015). The National Water Quality Monitoring Council (NWQMC) website, for example, has over 350 volunteer monitoring groups registered across the United States in 2018 (Baalbaki et al., 2019). A more recent study highlighted that citizen science will play an increasingly important role in promoting freshwater research, enhancing public understanding of the need to protect aquatic ecosystems, and engaging local communities and stakeholders in freshwater resource management (Metcalfe et al., 2022).

However, several research gaps persist. First, compared to the long history and prosperity of citizen science development in Europe and the United States, little research has been conducted in developing countries, including China (Baalbaki et al., 2019). This can be attributed to multiple barriers, such as the late commencement of citizen science initiatives in China, low participation levels, and issues related to data quality control. Consequently, the cooperation between Chinese scientists and the public is limited to a few citizen-based environmental projects, mainly focusing on bird and plant monitoring. Nevertheless, with growing concerns over environmental issues and the increasing influence of big data and social media in China, a new era of citizen science is emerging in China (Zhang et al., 2013). For example, a recent study conducted by Wu et al. (2022) revealed that most of the citizen science projects in China aiming to improve water quality are still ongoing, indicating the great potential of the citizen science approach for water monitoring in the country.

Second, a global review of citizen science projects related to water quality measurement over the past 20 years (Baalbaki et al., 2019) reveals a significant focus on chemical–physical parameters, such as nutrients, water turbidity, and temperature, with very few addressing waterborne pathogens. Among these waterborne pathogen assessments, coliforms – particularly *Escherichia coli* (*E. coli*) – are predominantly used as indicator organisms for assessing microbial contamination risk in water quality monitoring, both in China and internationally (Pandey et al., 2014; Feleni et al., 2025; Oon et al., 2023). However, the efficacy of indicator organisms in representing the potential presence of pathogens in water resources is still a subject of ongoing debate (Pandey et al., 2014; Motlagh and Yang, 2019; Richiardi et al., 2023). Specifically, a study suggests that China should incorporate additional microorganisms as alternative indicators of contamination to improve its water quality management (Wen et al., 2020). Therefore, it is necessary to assess microbial community compositions and pathogens in drinking water holistically.

Third, traditional microbiological monitoring of drinking water generally relies on culture-based methods, such as the heterotrophic plate counts (HPC) of specific microbes (Allen et al., 2004; Garner et al., 2021). However, these culture-dependent monitoring methods can only account for a tiny fraction (<1%) of the drinking water microbiome (Garner et al., 2021; Roeselers et al., 2015; van der Wielen and Kooij, 2010). To overcome this limitation, this study employed a culture-independent approach: microbial 16S rRNA gene metabarcoding. This method enabled a holistic survey of the microbial communities in the water samples (Garner et al., 2021; Ramirez et al., 2018; Thompson et al., 2017).

To the best of our knowledge, this study is among the first to employ a citizen science approach in collecting microbiome samples from household drinking water (i.e., tap water) across various regions in China, utilizing a simple yet cost-effective methodology. Following the protocol developed by this study, citizen scientists (college student volunteers) collected 50 tap water DNA samples from households across 31 administrative regions within 19 provinces and regions of China from December 2020 to August 2021. This dataset also includes several opportunistic samples collected shortly after extreme weather events, including the 2021 Henan Floods and Typhoon In-Fa Landfall. Microbial DNA was successfully extracted from these low-biomass tap water samples, and high-throughput sequencing on 16S rRNA gene amplicons was performed. This allowed us to characterize the total drinking water microbiome and identify potential waterborne pathogens.

## 2 Materials and methods

### 2.1 Sample collection

#### 2.1.1 Sites and participants

**Figure 1:**
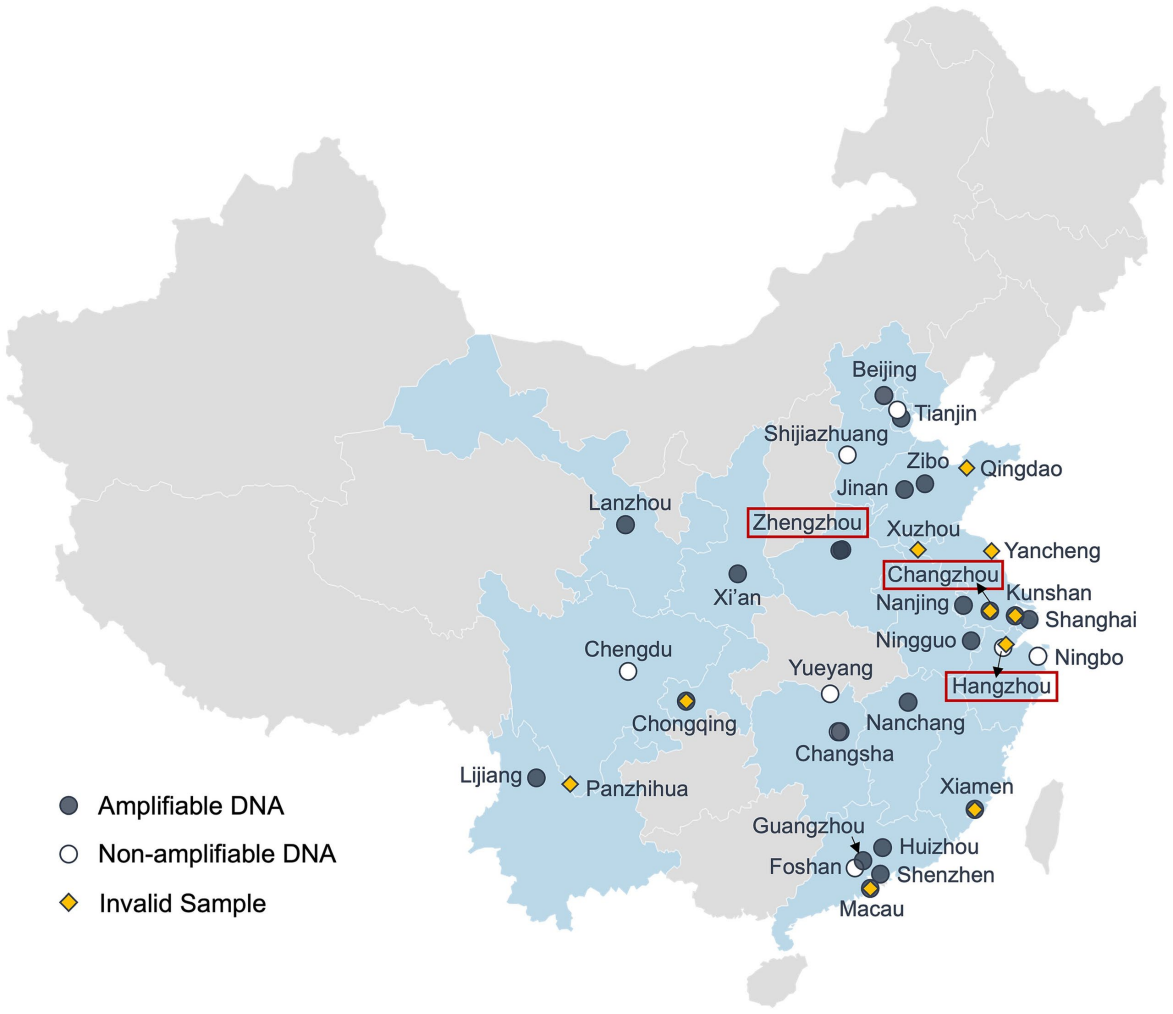
Geographical locations of sampling sites. Map showing the geographic locations of the citizen science samples and the PCR amplification results (Software: Datawrapper). Regions with samples are shaded in light blue. Locations with pre-and/or post-weather samples are labeled in red boxes. Black dots denote tap water samples showing positive PCR signals (amplifiable DNA), white dots indicate negative PCR signals (non-amplifiable DNA), and yellow diamonds represent samples that were collected but did not pass quality control. Some locations were sampled multiple times at different time points.

The sampling sites (Figure 1) were determined based on the coverage area (i.e., to include as many cities as possible) and the possibility of recruiting undergraduate student volunteers from the Duke Kunshan University (DKU) community. Due to the distribution of available volunteers, samples were mostly collected from central and eastern China (latitude: ~22°N–40°N; longitude: ~100°E–122°E), including Beijing, Shandong Province, Jiangsu Province, Guangdong Province, etc.

All student volunteers were recruited after a simple screening process. First, their relevant experiences (e.g., majors) were considered, and those with natural science majors and lab experience were preferred. Besides, ideal volunteers would go home or go on a trip to any place(s) in China during the sampling period. Communication between volunteers and investigators is a key component of this citizen science approach to ensure the quality of the samples as much as possible. Throughout the sampling period, volunteers could easily contact the research team via WeChat whenever issues or questions arose.

#### 2.1.2 Preparation, toolkit, and training

A standardized DNA sampling protocol was developed based on Buxton et al.’s (2018) research on citizen science methods, as volunteers in this study performed highly similar tasks (i.e., water sample collection and filtration) to those in their research. A detailed version of the protocol is provided in the Supplementary file 3 “Citizen Science Sampling Protocol and Materials” (CS 1). Two innovative aspects of this protocol are: (a) the easy-to-use and low-cost Corning syringe filters (instead of expensive pumps and Sterivex filter cartridges) were adopted for water sampling, and (b) the disinfection procedure was emphasized by listing all the possible exposed objects and surface during the entire sampling process. Briefly, it is advised to sample on the day or at most one day before shipping the sample. Volunteers first put on gloves and disinfect their hands, as well as everything they may touch during the operation, using disinfectant wipes (Brand: Mian Zhi Run, 75% Ethanol and RO purified water). Then, a sterilized 1 L stand-up bag with sodium thiosulfate to remove residual chlorine was unsealed and filled with 1 L tap water. A disposable, sterile 50 mL syringe was used to pass the sample water through a sterile Corning syringe filter unit (CLS431229, 0.20 μm pore size, 28 mm diameter) and was refilled until 1 L of water had been filtered or until the filter unit became blocked. Afterward, a syringe of air was pushed through the filter unit to reduce the amount of residual water in the sealed filter unit. The filter unit was then sealed in a Ziplock bag and stored frozen in a household freezer before being transported to the laboratory (DKU Environmental Research Lab in Kunshan). For transportation, the protocol requires burying the filter unit sample among four to five reusable blue ice packs in a Styrofoam box. Depending on the distance between the sampling site and Kunshan, along with the student’s mode of travel (i.e., same-day flights or high-speed train), samples were either delivered via next-day express delivery service or personally carried to the laboratory by volunteers.

**Figure 2:**
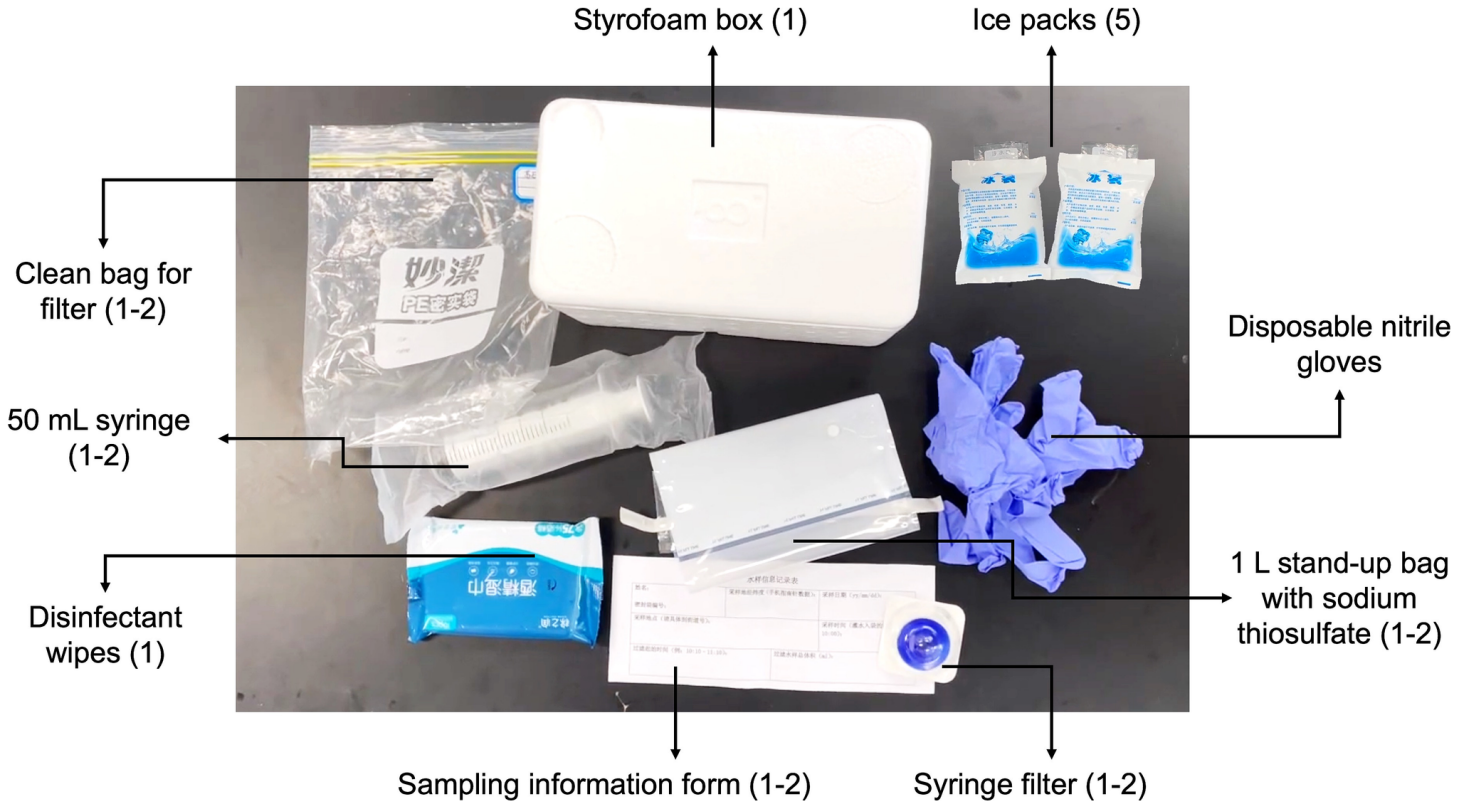
The sampling toolkit.

**Figure 3:**
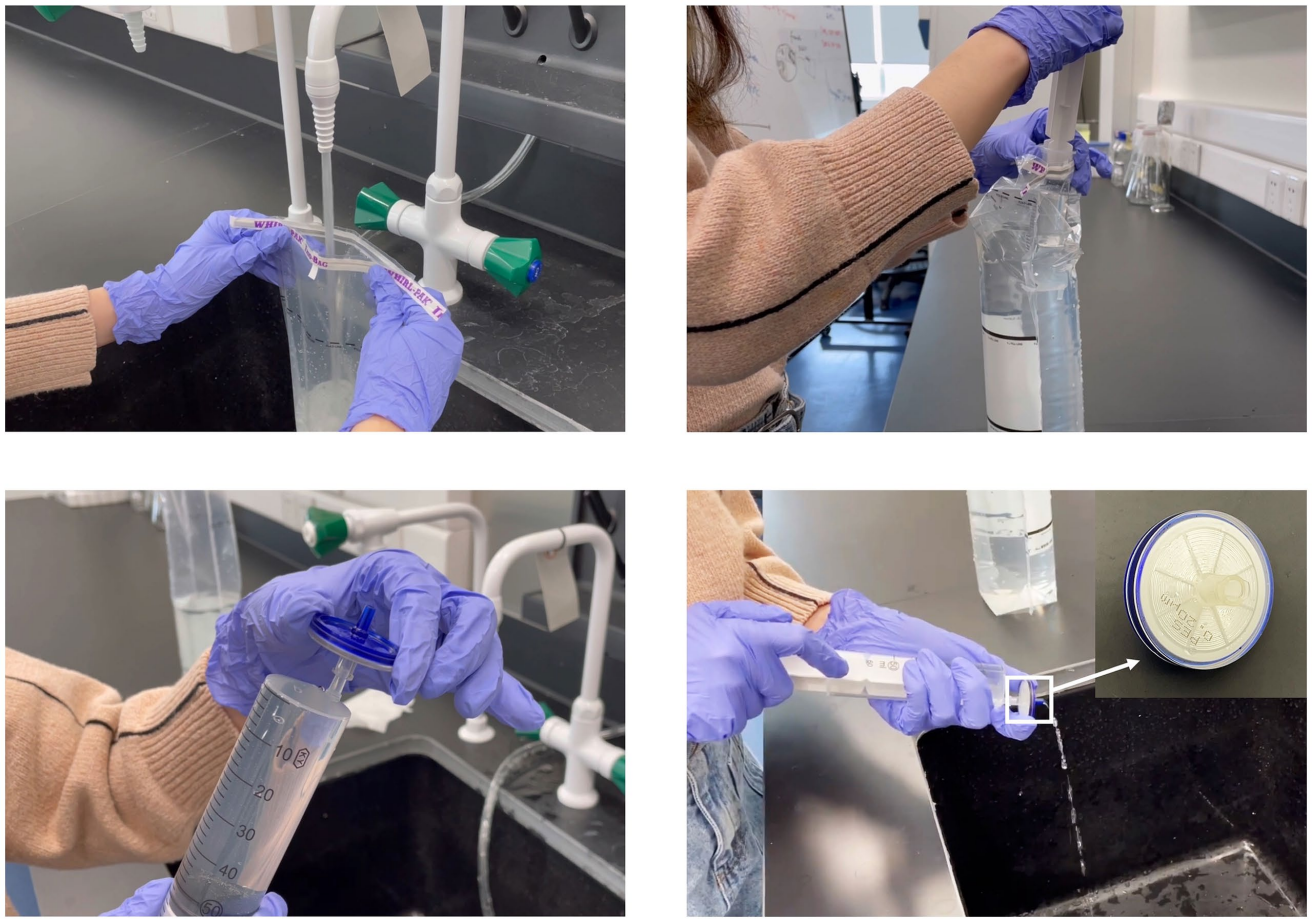
Screenshots from the demonstration video showing the sampling process and the syringe filter.

To further clarify the procedures and reduce variability in sample collection, an 11-min video tutorial (720p resolution) was created to provide volunteers with a visual guide on the toolkit and procedures for sample collection, filtration, storage, and shipping (Figures 2, 3 and CS 2). Furthermore, in-person training sessions were offered at the DKU Environmental Research Lab for available volunteers, following the demonstration model of Willis et al. (2018). A compact sampling kit was distributed to each volunteer prior to their trip (Figure 2). Each kit (in a 20.0 × 13.0 × 12.5 cm Styrofoam box) contained at least one set of all tools mentioned in the protocol and a sampling information form (CS 1 & 3) adapted from Buxton et al.’s (2018). This form collected information such as volunteer names, sampling time, and geographic coordinates of the sampling site obtained from cell phones, among other details. Alongside the form, volunteers were requested to label the Ziploc bag containing the sample with their names for convenient tracking of the sample during the data collection stage. Upon receipt in the lab, each sample was associated with an anonymous ID number, and any data containing identifiers was securely removed, ensuring that no specific identity-related information was present during data processing.

An online information packet was compiled and shared with each volunteer, which included a brief introduction to the research project, a sampling kit, and a sampling protocol, as well as links to the video tutorial and the electronic sampling information form (CS 3).

#### 2.1.3 Sample collection

The sample collection, conducted by student volunteers, took place from December 2020 to August 2021. At each sampling site, one to four samples were collected. For sites with more than one sample, all valid samples were included to represent the site’s microbiome. With the assistance of 25 volunteers, 50 household drinking water samples were collected from 31 administrative regions spanning 19 provinces and regions in China (Table 1).

**Table 1:** Sample information.

Sample ID	Sampling site (city, province or special administrative region)	Sampling date	Latitude	Longitude	Note
Valid samples					
*Positive signal*					
Beijing_0620	Beijing	2021/06/20	40.0	116.5	
Tianjin_0715	Tianjin	2021/07/15	39.1	117.2	
Zibo_0502	Zibo, Shandong	2021/05/02	36.8	118.0	
Jinan_0219	Jinan, Shandong	2021/02/19	36.6	117.1	
Lanzhou_0822	Lanzhou, Gansu	2021/08/22	36.1	103.8	
Zhengzhou_0219	Zhengzhou, Henan	2021/02/19	34.8	113.8	
Zhengzhou_0729	Zhengzhou, Henan	2021/07/29	34.8	113.8	Excluded
Zhengzhou_0723	Zhengzhou, Henan	2021/07/23	34.8	113.7	Excluded
Zhengzhou_0802	Zhengzhou, Henan	2021/08/02	34.8	113.7	
Xi’an_0217	Xi’an, Shaanxi	2021/02/17	34.3	109.0	
Nanjing_0219	Nanjing, Jiangsu	2021/02/19	32.1	118.7	
Nanjing_0717	Nanjing, Jiangsu	2021/07/17	32.1	118.7	Excluded
Changzhou_0220	Changzhou, Jiangsu	2021/02/20	31.8	119.9	
Changzhou_0712	Changzhou, Jiangsu	2021/07/12	31.8	119.9	
Changzhou_0728	Changzhou, Jiangsu	2021/07/28	31.8	119.9	
Kunshan_0524A	Kunshan, Jiangsu	2021/05/24	31.4	120.9	
Kunshan_0524B	Kunshan, Jiangsu	2021/05/24	31.4	120.9	Excluded
Shanghai_0411	Shanghai	2021/04/11	31.2	121.5	
Shanghai_0710	Shanghai	2021/07/10	31.2	121.5	Excluded
Ningguo_0217	Ningguo, Anhui	2021/02/17	30.6	119.0	
Chongqing_0715	Chongqing	2021/07/15	29.6	106.5	
Nanchang_0210	Nanchang, Jiangxi	2021/02/10	28.8	116.0	
Changsha_0801	Changsha, Hunan	2021/08/01	28.1	112.9	
Lijiang_0722	Lijiang, Yunnan	2021/07/22	26.9	100.2	
Xiamen_0504	Xiamen, Fujian	2021/05/04	24.6	118.3	Excluded
Huizhou_0219	Huizhou, Guangdong	2021/02/19	23.6	114.1	
Guangzhou_0220	Guangzhou, Guangdong	2021/02/20	23.1	113.3	
Shenzhen_0412	Shenzhen, Guangdong	2021/04/12	22.6	114.0	Excluded
Macau_0524	Macau	2021/05/24	22.1	113.6	
*Negative signal*					
Beijing_0127	Beijing	2021/01/27	40.0	116.5	
Tianjin_0327	Tianjin	2021/03/27	39.4	117.0	
Tianjin_0606	Tianjin	2021/06/06	39.4	117.0	
Shijiazhuang_0505	Shijiazhuang, Hebei	2021/05/05	38.0	114.4	
Chengdu_0219	Chengdu, Sichuan	2021/02/19	30.8	104.0	
Hangzhou_0716	Hangzhou, Zhejiang	2021/07/16	30.3	120.2	
Hangzhou_0727	Hangzhou, Zhejiang	2021/07/27	30.3	120.2	
Ningbo_0218	Ningbo, Zhejiang	2021/02/18	29.8	121.6	
Yueyang_0213	Yueyang, Hunan	2021/02/13	29.5	112.6	
Changsha_0213	Changsha, Hunan	2021/02/13	28.1	113.0	
Foshan_0219	Foshan, Guangdong	2021/02/19	23.0	113.1	
Invalid sample					
Macau	Macau	N/A	N/A	N/A	Excluded
Qingdao_0317	Qingdao, Shandong	2021/03/17	36.8	120.0	Excluded
Xuzhou_0219	Xuzhou, Jiangsu	2021/02/19	34.2	117.2	Excluded
Yancheng_0218	Yancheng, Jiangsu	2021/02/18	33.8	120.3	Excluded
Changzhou_1231	Changzhou, Jiangsu	2020/12/31	31.8	119.9	Excluded
Kunshan_0402	Kunshan, Jiangsu	2021/04/02	31.4	120.9	Excluded
Hangzhou_1228	Hangzhou, Zhejiang	2020/12/28	30.4	120.3	Excluded
Chongqing_0317	Chongqing	2021/03/17	29.6	106.5	Excluded
Panzhihua_0220	Panzhihua, Sichuan	2021/02/20	26.6	101.7	Excluded
Xiamen_0327	Xiamen, Fujian	2021/03/27	24.6	118.3	Excluded

During this study, two extreme weather events were captured by opportunistic sampling. Tap water samples were collected by volunteers from Changzhou, Jiangsu Province, and Hangzhou, Zhejiang Province from the same households before and after the landfall of Typhoon In-Fa (July 22–31, 2021 in China), and from Zhengzhou, Henan Province following an extremely destructive flood event (2021 Henan Floods). The samples collected after those two rainfall events are classified as “*post-weather samples*” (*n* = 5), while the rest are designated as “*normal samples*” (*n* = 45).

Typhoon In-Fa was a Category 2 typhoon (SSHWS), which was the second-wettest tropical cyclone ever recorded in China. As a tropical storm, it consecutively hit Putuo District of Zhoushan and Pinghu in Zhejiang Province on July 25 and 26, respectively. Typhoon In-Fa passed near Hangzhou from July 25 to 26 as a typhoon and Changzhou from July 26 to 27 as a tropical storm (NMC-Typhoon, 2016). On the other hand, the 2021 Henan Floods in Zhengzhou (July 17–23, 2021) was indirectly influenced by Typhoon In-Fa. From July 19 to 21, Zhengzhou suddenly encountered the most severe rainstorm of the last 50 years which led to extremely severe urban inland inundation, floods, and landslides (Chen, 2021), resulting in 16 million hectares of submerged agricultural land and direct economic damages amounting to $20.69 billion.

In areas affected by the typhoon landfall, student volunteers were instructed to sample tap water from the same household both before and after the typhoon within a one-week interval. As for the case of Zhengzhou, however, pre-flood sampling was not possible due to the unexpected onset of extreme rainfall and flooding (NMC, 2021). Despite this, we successfully coordinated with and supplied the tap water sampling kits to two volunteers residing in Zhengzhou—one in a severely affected area and the other in a moderately affected region—during the flooding event. They were instructed to collect three to four tap water samples on different days throughout the week following the flood.

### 2.2 DNA extraction, PCR amplification, and bacterial 16S metabarcoding

At the DKU Environmental Research Lab, all samples were stored in a −80°C freezer prior to processing. For each sample, the filter membrane was removed from the sealed syringe filter unit and cut into eight strips using a sterile razor blade on a disposable petri dish. DNA extraction was then conducted using the Qiagen DNeasy Mini Kit, following the manufacturer’s instructions, with an additional bead-beating step to enhance extraction efficiency. In brief, the filter strips were placed into a 2 mL microcentrifuge tube, where 0.2 g of 0.1 mm Zr beads and 400 microliters of lysis buffer AP-1 were added. The mixture was then beaten at 2,000 rpm for 5 min.

For PCR amplification, the V4 region of the 16S rRNA gene was amplified by the universal primer pairs 515F (5′-GTGCCAGCMGCCGCGGTAA-3′) and 805R (5′-GACTACNVGGGTATCTAAT-3′) with dual barcode index and heterogenous spacers (Lin et al., 2019; Kozich et al., 2013). KAPA HiFi PCR Kit and the manufacturer’s protocol were adopted. All PCR reactions were performed in triplicates with 25 μL of each reaction mixture. Agarose gel electrophoresis was then performed to visualize amplicon fragments, and PCR products were purified using the QIAquick PCR Purification Kit (Qiagen) following the manufacturer’s instructions. The purified PCR amplicons from each sample were dissolved in 30 μL of elution buffer and stored in a −80°C freezer.

Finally, the concentration of the purified PCR amplicons was measured using a Qubit™ 4 Fluorometer. Equimolar amounts of purified amplicons were pooled together and sent to *Genewiz* in Suzhou for an Illumina Miseq (250 PE) sequencing run.

### 2.3 Data analysis

#### 2.3.1 Sequence processing

Paired-end sequencing reads with dual indices were demultiplexed and then trimmed to remove barcodes and primers using *Cutadapt* (Martin, 2011). The resulting reads were then further processed using the *DADA2 (v1.16)* pipeline (Callahan et al., 2016). Specifically, using the embedded functions in *DADA2*, quality filtering was performed before merging paired-end sequencing reads. After chimera checking, the Amplicon Sequence Variant (ASV) was identified, and the abundance table for each sample was constructed. Finally, the “assignTaxonomy” function in *DADA2* was used to assign taxonomy to each ASV based on the Silva r138 reference database (DOI 10.5281/zenodo.4587955).

#### 2.3.2 Microbial community structure and potential waterborne bacterial pathogens

The relative abundance (RA) of all ASVs in each sample was calculated and the following analyses of microbial compositions were based on RA. The bacteria genera in the dataset containing pathogenic species were selected based on *Aquatic Pollution: An Introductory Text* (Laws, 2017) and “Guidelines for Drinking-water Quality (4th Edition)” by WHO (2017). Subsequently, the taxonomy of the resulting ASVs was further validated using the BLAST+ tool (Camacho et al., 2009) against the NCBI database in February 2022. Based on our criteria, only BLAST results with percent identity (p-ident) > 97% (Johnson et al., 2019), expect value (E-value) < 10e-100 (Vej, 2007), and query cover equals 100% were considered reliable results.

Subsequently, all potential pathogenic genera and species were grouped into two categories based on their occurrence in the drinking water samples. Those detected in more than 30% of all samples were categorized as “common pathogens” while the rest were referred to as “rare pathogens.”

#### 2.3.3 Statistical analyses

All statistical analyses were performed in R (version 4.1.2 and 4.2.3), and a *p*-value threshold of 0.05 was considered statistically significant. The R scripts and accompanying data files can be accessed via the following GitHub repository: https://github.com/YajuanLin/citizen-science-pathogen.

Alpha diversity was calculated at the ASV level to further describe the microbial communities in the samples (Andermann et al., 2022). Each library was resampled to an equal depth, and Chao1, Fisher, Shannon, and Simpson diversity indices were then calculated from the observed read counts of ASVs using the “Phyloseq” package (version 1.38.0) in R (McMurdie and Holmes, 2013). The Shannon and Simpson indices, including both ASV richness and evenness, were computed due to their reduced sensitivity to differences in sample depth (Haegeman et al., 2013; Preheim et al., 2013).

To explore potential ecological relationships among key microbial groups in tap water and identify possible indicator taxa or co-occurrence patterns, we assessed linear associations using Spearman’s rank correlation coefficients between the RA of individual potential pathogens, the total RA of those pathogens, and alpha diversity indices. The correlation analysis was performed using the *cor() function*. The corresponding *p* values and confidence intervals were computed with *cor.mtest()*. The matrix with significance level codes was visualized with *corrplot.mixed()*.

## 3 Results and discussion

### 3.1 Sample validity and PCR signals

Out of the 50 drinking water samples, 40 passed our quality control and they were collected from 27 administrative regions across China (Table 1). Ten samples were excluded from the study because of either lab processing errors or improper handling during shipping and/or storage (e.g., all ice packs melt), which may have compromised DNA quality. Among the 40 valid samples, 29 samples showed positive PCR signals which were collected from 16 cities in 10 provinces, 4 municipalities, and Macau (Figure 1). Notably, all three tap water samples collected in Zhejiang Province, including the post-typhoon Hangzhou sample showed no PCR signal, suggesting that microbial contamination was below the detection limit of the PCR approach for the 1-liter water samples. This result may reflect the high quality of source water in Zhejiang Province (Han et al., 2020) and effective drinking water treatment in developed cities such as Hangzhou and Ningbo. Alternatively, factors such as new/clean plumbing systems, suitable plumbing materials that do not support the growth of microorganisms, and shorter water stagnation time in the plumbing could also account for the absence of detectable microbial DNA (Ley et al., 2020; Ji et al., 2015; Ling et al., 2018).

### 3.2 Citizen science sampling approach

This study is among the few investigations on the tap water microbiome conducted through citizen science, which can serve as a proof of concept for national-scale microbiological monitoring of tap water using citizen science and show its competitive advantages compared to non-citizen science sampling methods.

Firstly, the study demonstrates the cost-effectiveness and extensive coverage of the approach across China. With decentralized volunteer participation and affordable sampling kits, the sampling sites spanned 31 regions and various seasons, ensuring spatial and temporal diversity. It is worth mentioning that the use of Corning^®^ syringe filters (~$2.5–4/unit) (Corning, 2023) reduced costs by 1 to 1.5 times compared to the traditional MilliporeSigma^®^ Sterivex filters (~$8–13/unit) commonly used for aquatic microbiome sampling (Millipore Sigma, 2023). Also, our DNA extraction protocol was specifically optimized for this low-cost filter. Additionally, this citizen science sampling approach significantly reduces travel and personnel expenses compared to traditional fieldwork methods. While the lack of strict oversight during the volunteers’ sample collection is a limitation, the high validity, with 40 out of 50 drinking water samples passing quality control, and the consistency in results discussed in the following sections support the approach’s applicability and reliability.

Furthermore, due to the flexibility of this sampling approach, we conducted timely opportunistic sampling of extreme weather events—specifically, Typhoon In-Fa and 2021 Henan Floods—by leveraging local volunteers in Hangzhou, Changzhou, and Zhengzhou. This citizen science approach proved to be particularly fast-responding and effective given the limited funding and the urgent nature of these events. Additionally, the sample storage tests (Supplementary Text 1.1) confirmed that maintaining samples for 7 days at −18°C did not generate false positives in the absence of bacteria during collection, ensuring accurate pathogen identification and reliability of using household freezer for storage, thereby enhancing the flexibility and feasibility of the citizen science sampling approach.

### 3.3 Sequence reads, ASVs, and taxonomy classification

The DNA of microorganisms in 29 household drinking water samples were successfully extracted, PCR amplified with 16S primers, and submitted for Illumina sequencing. Following the demultiplexing process, five samples did not yield significant reads (all < 50 per library) and were excluded from the dataset. In addition, two samples collected after the 2021 Henan Floods were excluded after quality filtering and chimera checking due to having relatively low reads (<3,000 per library). Analysis of the sequencing results indicated that samples with low read counts were primarily associated with specific primer sets, suggesting that the reduced output was likely due to primer efficiency or compatibility issues rather than issues with DNA quality. As a result, the 16S rRNA gene amplicon dataset in this study comprised a total of 22 samples.

In DADA2 quality filtering, the proportions of output and input read for 13 (59.1%) samples were lower than 50%, indicating low-quality raw DNA reads. This may be due to (1) potential degradation of DNA samples during sampling, shipping, or DNA processing; and (2) residual DNA from dead bacteria cells, which are harmless to humans. After quality filtering and chimera removal, the sequencing reads per sample ranged from 9,794 to 150,656, with an average of 60,884. From the resulting dataset, 7,635 ASVs were identified (the most abundant ASVs are provided in Supplementary Text 1.2 and Supplementary Table 2.1).

### 3.4 Microbial community structure

Fifty two microbial phyla including 46 bacterial phyla and 6 archaeal phyla were detected in the household drinking water samples (*n* = 22). In all samples, 9 bacterial phyla and 1 archaeal phylum accounted for over 90.7% of total taxonomically assigned reads at the phylum level (Table 2). All 10 phyla are both abundant and prevalent (i.e., occur in 91–100% of all samples), indicating a relatively even microbial distribution pattern at the phylum level in the samples. The five most dominant bacterial phyla were *Proteobacteria* (mean percentage ± SD: 55.0% ± 19.8%), *Planctomycetota* (10.5% ± 7.9%), *Acidobacteriota* (7.0% ± 6.0%), *Actinobacteria* (5.9% ± 6.6%), and *Cyanobacteria* (4.7% ± 3.5%), comparable to a previous study in China (Han et al., 2020). Four of them were reported to be tolerant to water treatment and distribution processes, except for *Acidobacteriota* (Han et al., 2020; Godinho et al., 2024). Interestingly, this bacterial composition differs from the primary bacterial assemblages revealed by several highly cited studies conducted in other countries, including the United States and Portugal, which predominantly feature *Proteobacteria*, *Actinobacteria*, and *Bacteroidetes* (Hull et al., 2017; Pinto et al., 2012; Ivone et al., 2013). This may imply a unique microbiome intrinsic to China’s drinking water systems.

**Table 2:** Number of sequences, ASVs, and genera for the top 10 phyla in the water samples.

Phylum	Sequence^a^	ASV	Genus
*Proteobacteria*	697,642 (52.3%)	2,693	281
*Planctomycetota*	164,487 (12.3%)	998	23
*Actinobacteriota*	82,262 (6.2%)	279	56
*Acidobacteriota*	77,415 (5.8%)	296	17
*Cyanobacteria*	72,219 (5.4%)	402	25
*Crenarchaeota*	43,624 (3.3%)	31	5
*Bacteroidota*	38,869 (2.9%)	603	61
*Verrucomicrobiota*	33,769 (2.5%)	411	27
*Bdellovibrionota*	31,056 (2.3%)	400	8
*Desulfobacterota*	21,608 (1.6%)	59	11
Others	70,371 (5.3%)	1,209	93
N. A.	6,133	254	/

Those three represent the coverage, diversity, and the genus spectrum of the microbial community in the water samples, respectively.

a The percentage of sequences was calculated as the number of phylum sequences in the total assigned sequences at the phylum level, which was 1,333,322.

**Figure 4:**
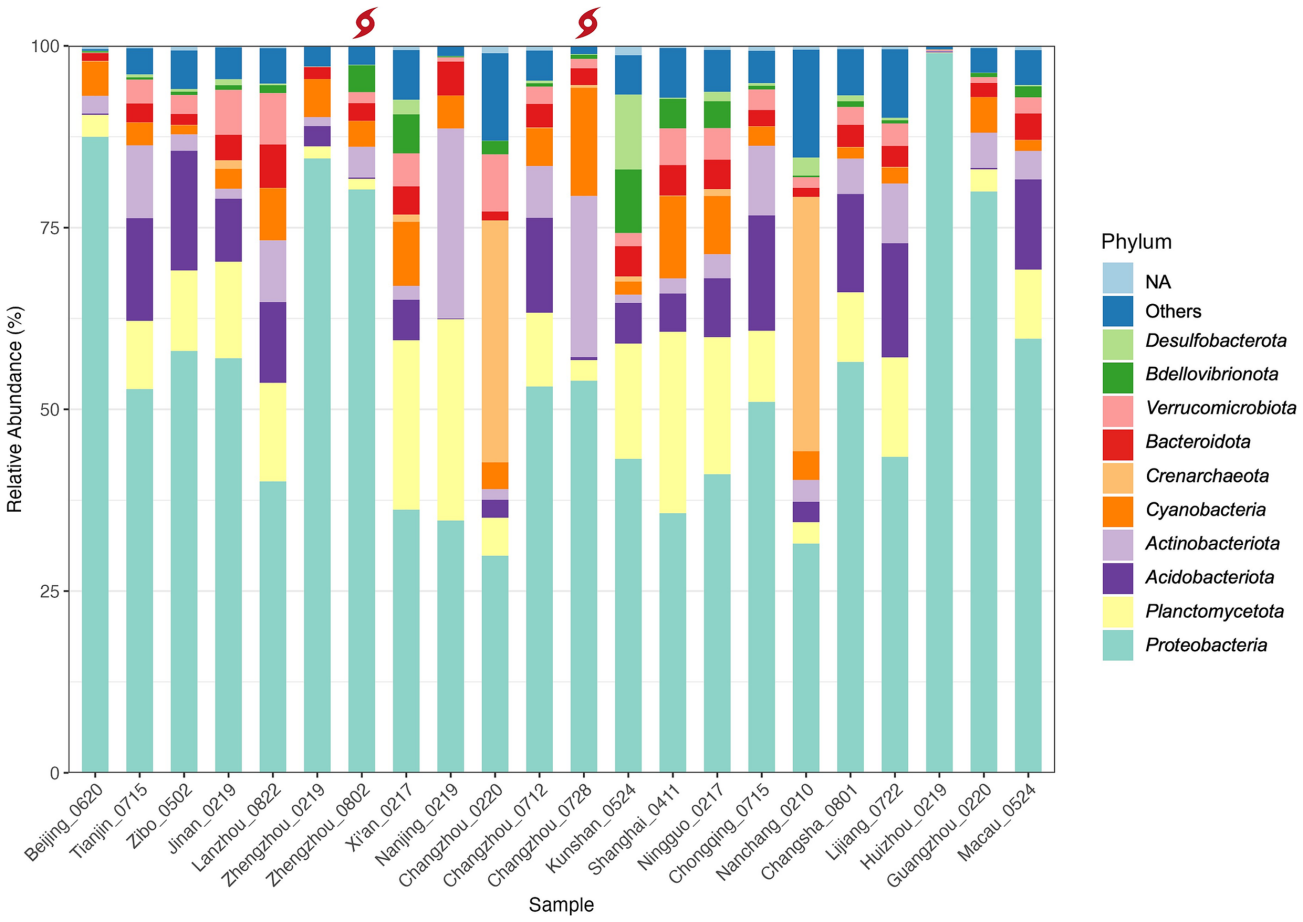
Taxonomic composition and relative abundance of microbiota in the sampled household drinking water in China at the phylum level. The red typhon icon indicates samples collected after extreme rainfall events. Samples are arranged in order of latitude. Only the top 10 most abundant phyla (listed bottom to top in the legend) and unassigned (NA) phyla are shown; all remaining phyla are grouped under “Others”.

The phylum-level taxonomic composition for each sample is detailed in Figure 4 and Supplementary Table 2.2 (the most abundant classes are summarized in Table 3). In this study, *Proteobacteria* (classes α*-*and γ*-Proteobacteria*) was the most predominant phylum in 20 samples and the second most prevalent in others. It accounts for 29.9–99.0% of the reads in each sample, with the highest relative abundance (RA) detected in Huizhou (Huizhou_0219). Among all the samples, *Sphingomonas* was the most abundant genus of *Proteobacteria*. This result was consistent with a previous study (Han et al., 2020) which found *Proteobacteria* to be dominant in tap water collected mainly from central and eastern China, and the genus *Sphingomonas* grew during chlorination (Jia et al., 2015) or monochloramine treatment (Chiao et al., 2014).

**Table 3:** Top 10 classes in the normal samples (*n* = 20).

Class	Avg. relative abundance
*Alphaproteobacteria*	37.43%
*Gammaproteobacteria*	16.28%
*Planctomycetes*	7.97%
*Blastocatellia*	5.71%
*Actinobacteria*	4.24%
*Nitrososphaeria*	3.62%
*Bacteroidia*	2.64%
*Verrucomicrobiae*	2.20%
*Vampirivibrionia*	2.14%
*Cyanobacteriia*	1.76%
Other	13.19%
NA	2.81%

However, two samples, Changzhou_0220 (Changzhou, Feb. 20) and Nanchang_0210 (Nanchang, Feb. 10), were dominated by *Crenarchaeota*, a common archaeal phylum. Specifically, *Crenarchaeota* accounted for 33.3 and 35.0%, with the genus *Candidatus Nitrosotenuis* (32.0%) and *Candidatus Nitrosotalea* (34.9%) being most abundant in samples Changzhou_0220 and Nanchang_0210, respectively. This indicates that, in addition to a variety of bacteria, archaea can also grow in tap water, which is supported by studies that have detected the archaeal phylum *Crenarchaeota* in drinking water distribution systems and drinking water-related environments (Roeselers et al., 2015; Inkinen et al., 2021; Dai et al., 2020; Franca et al., 2015; Bautista-de los Santos et al., 2016). In particular, Inkinen et al. (2021) found a high abundance of archaeal reads from the genus *Candidatus Nitrosotenuis* and *Candidatus Nitrosotalea* in drinking water distribution systems supplying non-disinfected waters. This suggests that the disinfection processes of samples Changzhou_0220 and Nanchang_0210 may be less effective compared to others.

Interestingly, compared to the winter Changzhou sample Changzhou_0220, the pre-typhoon Changzhou_0712 and post-typhoon samples Changzhou_0728 collected from the same urban household in July were much more similar in overall composition at the phylum level, indicating a seasonal effect. However, the post-typhoon sample Changzhou_0728 exhibited higher levels of *Actinobacteria* (increased from 2.2 to 7.1%) and *Cyanobacteria* (increased from 5.2 to 14.9%), which are phyla containing potential waterborne pathogens and the species that produce cyanotoxins, respectively. Elevated levels of the pathogen *Mycobacterium* spp. (more details in Section 3.5) as well as toxin-producing *Cyanobacteria* spp. were observed in post-typhoon sample Changzhou_0728. Specifically for cyanobacteria, *Microcystis* spp. had a higher RA, while *Cylindrospermopsis* sp. and *Dolichospermum* sp. appeared after the typhoon event. Other toxic species of *Cyanobacteria*, including *Aphanizomenon* sp. and *Anabaena* sp., were detected in normal samples collected from Shanghai, Lanzhou, Xi’an, and other locations. Many *Cyanobacteria* spp. from those genera can produce a variety of cyanotoxins such as Microcystins and Cylindrospermopsin, which can cause liver and kidney damage and have potential carcinogenicity (EPA, 2022). Similarly, a substantial rise in the RA of *Cyanobacteria* was reported in treated water samples collected from a drinking water treatment plant in Jiangsu Province after Typhoon Lekima in August 2019 (*p* < 0.05) (Tang et al., 2021). In this study, although the RA of *Cyanobacteria* in the post-typhoon sample Changzhou_0728 remained detectable, it decreased to the pre-typhoon level by the third day after the typhoon event.

### 3.5 Potential pathogenic bacteria in drinking water microbiome

In total, six bacteria genera containing pathogenic species and three pathogenic species were detected in all the PCR positive samples (*n* = 22) (Table 4). Five genera and one species that occurred in more than 30% of the samples (7 samples) were categorized as common pathogens, while the rest were grouped into rare pathogens (Supplementary Table 2.3). It is worth noting that the overall pattern of pathogens in the normal samples remains unchanged when the two post-weather samples are included. This finding suggests that pathogen contamination in tap water could be a widespread phenomenon (Ley et al., 2020; Ma et al., 2022).

**Table 4:** Occurrence and mean relative abundance (RA) of major potential pathogens.

Pathogen species	Occurrence	Mean RA
*Mycobacterium* spp.	22 (100.0%)	2.67E-02
*Acinetobacter* spp.	22 (100.0%)	1.43E-02
*Legionella* spp.	22 (100%)	4.59E-03
*Brevundimonas* spp.	20 (90.9%)	6.12E-03
*Leptospira* spp.	11 (50.0%)	2.04E-04
*Escherichia coli*	8 (36.4%)	5.80E-04
*Bacillus* spp.	3 (13.6%)	2.01E-05
*Aeromonas hydrophila*	2 (9.1%)	4.76E-05
*Salmonella enterica*	1 (4.5%)	1.79E-04

**Figure 5:**
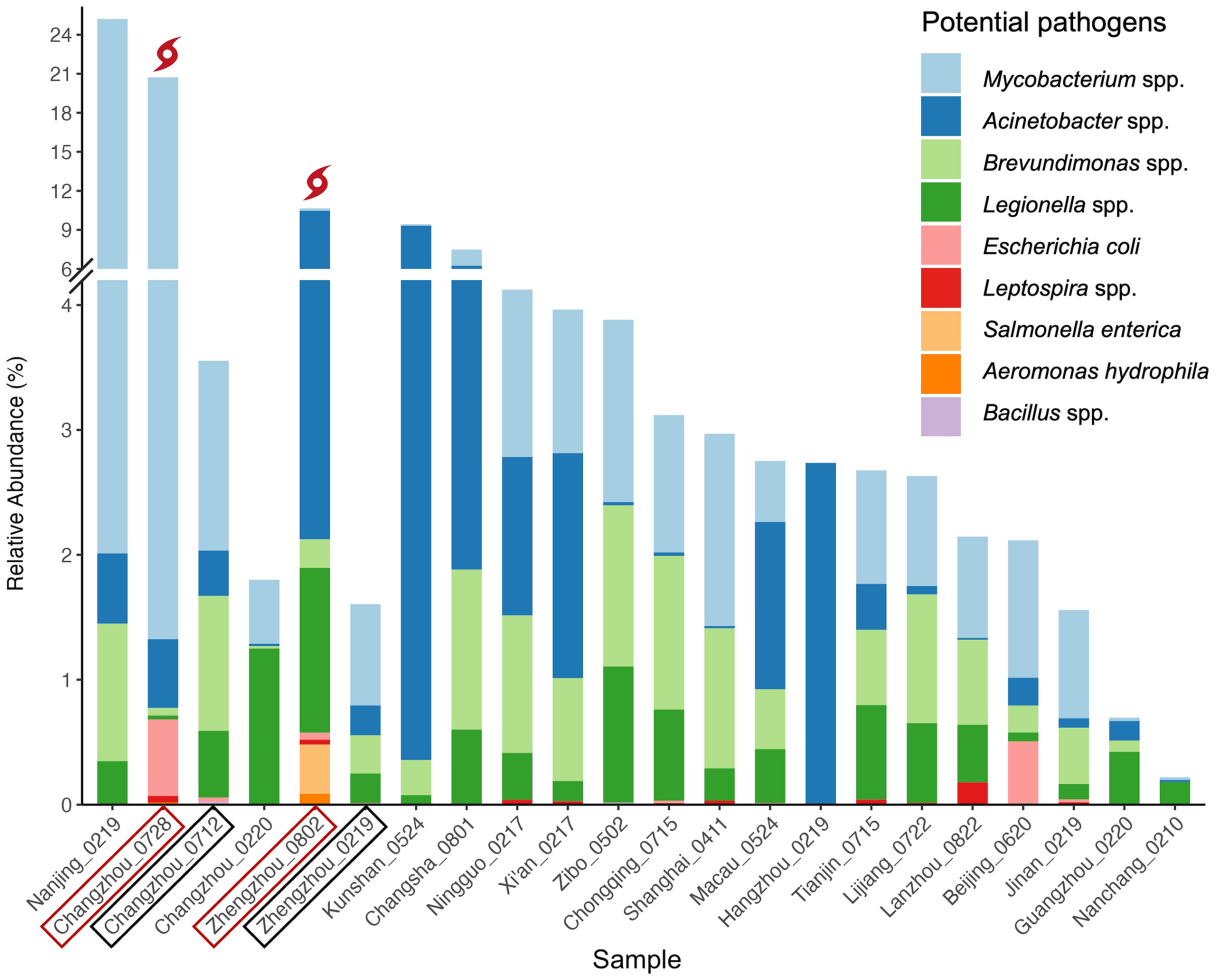
Relative abundance of potential bacterial pathogens. Pathogens in the figure legend are in the descending order of mean RA (from top to bottom). Samples are in the descending order of total RA of all pathogens in each sample (from left to right) except for locations with multiple samples. For these locations, pre-and post-weather samples are grouped together to facilitate comparison and are labeled in black boxes and red boxes, respectively.

The distribution of these potential pathogens within each water sample is detailed in Figure 5, and the BLAST+ results of the potential pathogenic ASVs are provided in Supplementary Table 2.4. The mean RA of common pathogens in all tap water samples ranged from 0.02% to 2.67% (normal samples: 0.02%–1.95%), while that of rare pathogens was extremely low. *Mycobacterium* spp. (mean RA 2.67%, including rainfall events)*, Acinetobacter* spp. (1.43%), and *Legionella* spp. (0.46%) occurred in all the samples while *Leptospira* spp. (0.02%) were found in half of the samples. Notably, *Escherichia coli* ASVs (0.06%) (E-value = 6e-132, Percent identity = 100%) were detected in 36.4% of the samples. In addition, two *Brevundimonas* species, *B. vesicularis* and *B. diminuta*, which are particularly recognized as emerging global opportunistic pathogens (Ryan and Pembroke, 2018), were detected in the majority of samples (90.9%). Compared to normal samples, three more pathogenic bacteria species were detected in the post-weather samples, including *Salmonella enterica*, *Brevundimonas diminuta*, and *Aeromonas hydrophila.*

*E. coli*, a common fecal indicator bacteria (WHO, 2017), was widely detected in this study. The tap water samples with the highest RA of *E. coli* were Changzhou_0728 (RA: 0.61%), Beijing_0620 (0.51%), Zhengzhou_0802 (0.05%), Changzhou_0712 (0.03%), and Jinan_0219 (0.02%). The elevated RA of *E. coli* in the two post-weather samples suggests that the contamination might be related to the extreme rainfall events. Notably, the proportion of *E. coli* in Beijing_0620 was dramatically higher than that of other normal samples. It is worth mentioning that traditional *E. coli* tests can be a generally reliable indicator of enteropathogenic serotypes in drinking water; however, potentially viable but non-culturable *E. coli* cells could result in underestimations of actual water contamination (Liu et al., 2008). Therefore, it is recommended to use PCR or quantitative PCR (qPCR) methods for the monitoring of *E. coli* (Wolf-Baca and Siedlecka, 2019).

Additionally, the RA of total *Legionella* spp. was most abundant in Zhengzhou_0802 (1.32%), Changzhou_0220 (1.25%), and Zibo_0502 (1.09%). Almost all species in the genera *Legionella* are thought to be potential human pathogens, but *L. pneumophila* (on Contaminant Candidate List 5 - CCL 5) is the leading cause of Legionnaires’ disease (pneumonia) and Pontiac fever (a milder infection) (WHO, 2017; EPA, 2023). Potential *L. pneumophila* ASVs were detected in 22.7% (Ramirez-Castillo et al., 2015) of the tap water samples. Among those samples, the highest *L. pneumophila* RA was observed in Zhengzhou_0219 (RA: 0.237%), followed by Macau_0524 (0.035%), Nanchang_0210 (0.019%), Xi’an_0217 (0.011%), and Shanghai_0411 (0.010%). Moreover, some other pathogenic species such as *L. oakridgensis* and *L. maceachernii* occurred in Tianjin_0715, Lanzhou_0822, Xi’an_0217, Lijiang_0722, and Zhengzhou_0802.

*Salmonella enterica* (ASV 3082, RA: 0.39%), a highly pathogenic species, was detected in the post-flood tap water microbiome from Zhengzhou (Zhengzhou_0802), despite its known susceptibility to disinfection. However, the potential health risks remain uncertain, as the severity of the disease depends on the serotype and host factors of *Salmonella* (WHO, 2017). Nonetheless, the presence of *Salmonella enterica* after the 2021 Henan Floods indicates potential contamination in the household drinking water after an extreme weather event. While this could suggest fecal contamination, alternative sources, such as residual contamination from washing raw meat in kitchen sinks, cannot be ruled out.

**Figure 6:**
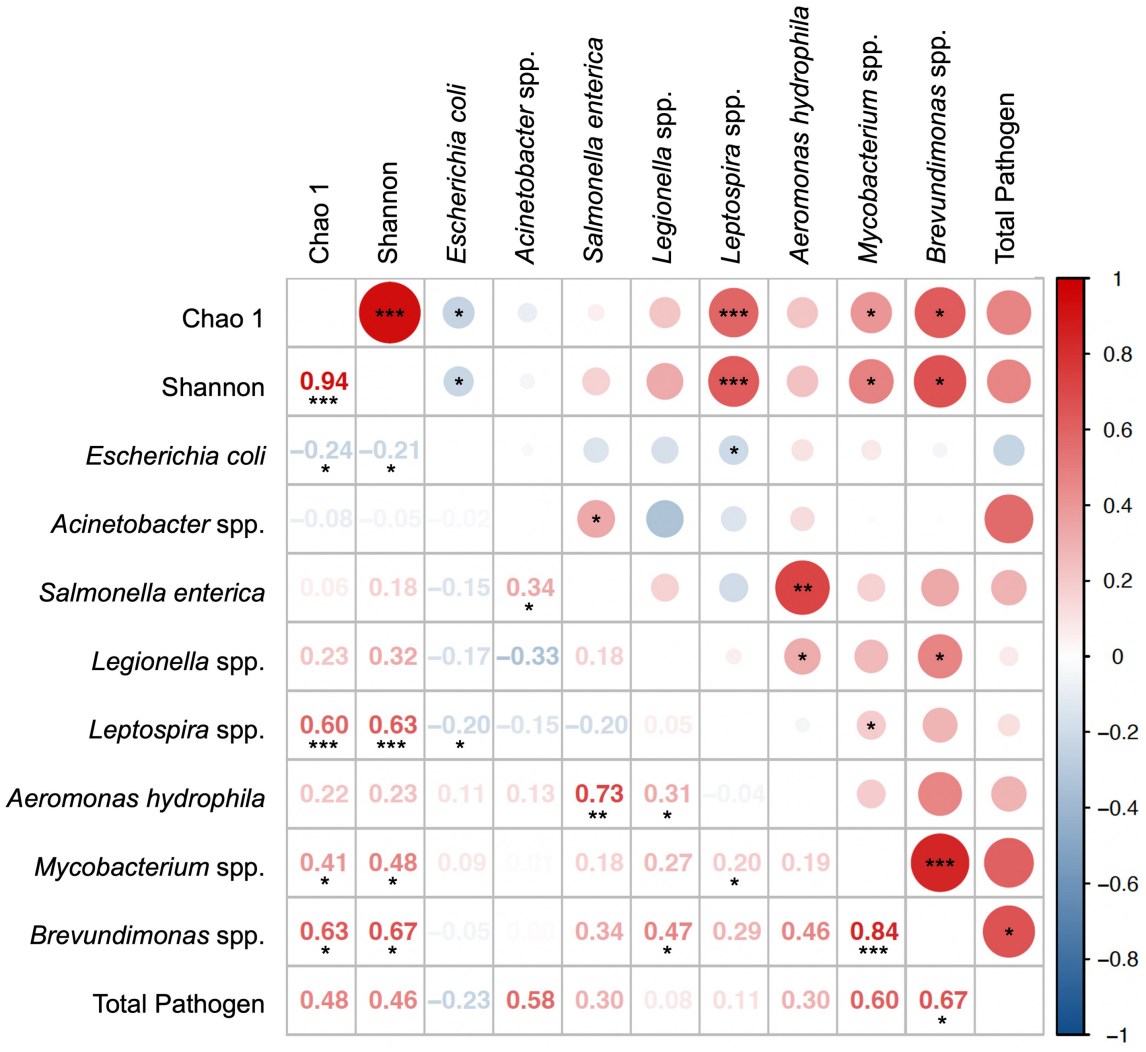
Spearman’s correlation of potential pathogens detected in normal samples (*n* = 20).*, **, *** denotes the significance level of 0.05, 0.01, and 0.001, respectively.

Spearman’s correlations between potential pathogens and total microbiome alpha diversity indexes (Chao1 and Shannon) are shown in Figure 6. When the influence of extreme rainfall events was excluded (*n* = 20), the RA of *Brevundimonas* spp. showed strong positive correlations with multiple pathogens and alpha diversity indexes (Supplementary Table 2.5), especially with *Mycobacterium* spp. (*r* = 0.84, *p* < 0.001) and the total RA of potential pathogens (*r* = 0.67, *p* < 0.05). These findings suggest that *Brevundimonas* spp. may serve as a useful ecological indicator of microbial risk in tap water systems.

Interestingly, we also observed that the presence of *E. coli* was significantly associated with lower alpha diversity of the microbial community (Chao1: *r* = −0.24, *p* < 0.05; Shannon: *r* = −0.21, *p* < 0.05). Reduced alpha diversity may reflect microbial imbalance or stress conditions that favor pathogen persistence. Understanding the relationship between indigenous water microbiomes and opportunistic or fecal pathogens, such as *E. coli,* could therefore provide insights into the ecological conditions that support pathogen survival in tap water.

While some correlations may be driven by low sample detection frequencies or shared habitat traits, the broader pattern highlights the value of co-occurrence analysis in generating hypotheses about ecological interactions or stress responses in tap water microbiomes. We acknowledge these are exploratory findings, and future studies incorporating environmental covariates (e.g., chlorine levels, pipe material, water age) would help validate these relationships.

### 3.6 Limitations and future work

The citizen science sampling procedure exhibits several limitations and could be improved in the future. First, to further minimize bias during the sample collection process, closer supervision of the volunteers (e.g., through cell phone video recording) and duplicate sample collection from the same location would be beneficial. Second, systematic time-series sampling from the same location is necessary to understand the temporal patterns of microbes. Additionally, to pinpoint contamination sources, future studies could sample water treatment system effluents to determine if contamination originates from treatment or pipeline issues. Moreover, collecting a comprehensive set of metadata (e.g., water temperature, nutrients, pH) associated with microbiome sampling will help reveal the environmental factors shaping the drinking water microbiome.

To improve the success rate of sample collection, it is essential to implement stricter controls on shipping temperatures (e.g., adding a reusable temperature logger to the sampling kit) and to collaborate with shipping companies. This collaboration will help reduce sample transportation costs, a critical factor in expanding the citizen science outreach beyond university affiliates. Meanwhile, developing an online platform could help disseminate the research results to the public, thereby promoting science education and citizen engagement.

## 4 Conclusion

Ensuring household drinking water safety is vital for public health due to the risks associated with microbial contamination. Combining citizen science sampling and culture-independent metabarcoding, this proof-of-concept study provided profiles of tap water microbiome and waterborne pathogens from various locations in China. This method, which extends beyond basic water collection and observation (e.g., water turbidity), suggests that well-structured collaborations between professional agencies and citizen science can effectively monitor water quality on a broad scale.

In this study, a total of 7,635 prokaryotic ASVs were detected in 40 household drinking water samples from 27 cities across 19 provinces and regions in China. Although based on a limited number of samples, the findings suggest that extreme weather events such as typhoons and floods may increase the presence of potential pathogens (e.g., *Escherichia coli*, *Salmonella enterica*) and toxin-producing cyanobacteria such as *Microcystis* in local tap water. This is particularly concerning in the current context of climate change, which may increase the frequency and intensity of such conditions. Additionally, this study highlights the valuable role that citizen science can play in advancing our understanding of environmental health risks and shaping public health policy.

## Data Availability

The original contributions presented in the study are publicly available. This data can be found here: https://www.ncbi.nlm.nih.gov/, PRJNA1292393.
